# A Historical and Epistemological Review of Type 1 Diabetes Mellitus

**DOI:** 10.3390/jcm14144923

**Published:** 2025-07-11

**Authors:** Eugenio Cavalli, Giuseppe Rosario Pietro Nicoletti, Ferdinando Nicoletti

**Affiliations:** Department of Biomedical and Biotechnological Sciences, University of Catania, 95123 Catania, Italy; eugeniocavalli9@hotmail.it (E.C.); giunicol01@gmail.com (G.R.P.N.)

**Keywords:** autoimmunity, biomedical epistemology, cytokines, disease paradigm shift, historical review, HLA class II, islet autoantibodies, immunotherapy, teplizumab, predictive Medicine

## Abstract

Over the past century, the understanding of type 1 diabetes mellitus (T1DM) has evolved significantly, transitioning from a fatal metabolic disorder to a well-characterized autoimmune disease. This review explores the historical developments and scientific milestones that have reshaped the perception of T1DM, highlighting key discoveries and shifts in medical paradigms. **Methods:** A comprehensive narrative review was conducted, examining literature spanning from ancient medical texts to contemporary research up to 2024. Emphasis was placed on pivotal moments such as the discovery of insulin in 1921, the recognition of autoimmune mechanisms in the 1970s, and recent advancements in immunotherapy. **Results:** The reclassification of T1DM as an autoimmune disease was supported from multiple lines of evidences including the presence of islet cell autoantibodies, the identification of lymphocytic infiltration in pancreatic islets, and the associations of the disease with certain HLA class II alleles. The development of animal models and large-scale cohort studies facilitated the establishment of disease staging and risk prediction models. Notably, the approval of immunotherapies like teplizumab underscores the translational impact of these scientific insights. **Conclusions:** The historical trajectory of T1DM exemplifies the dynamic nature of medical knowledge and the interplay between clinical observations and scientific research. Recognizing these developments enhances our comprehension of disease mechanisms and informs current approaches to diagnosis and treatment.

## 1. Introduction

The story of type 1 diabetes mellitus (T1DM) is not only a chronicle of medical discovery but also a case study in the evolution of scientific thought. For much of recorded history, diabetes was considered as a singular metabolic disorder a condition defined by emaciation, glycosuria, and a rapid course toward death. With the advent of insulin therapy in the early 20th century, T1DM became treatable with exogenous insulin but remained etiologically obscure. It took several decades, and the convergence of multiple lines of evidences, before T1DM could be reclassified as an autoimmune disease. This reclassification was not just terminological as this marked a change in underlying the way of understanding the pathogenesis of the disease and the exploitation of novel therapeutic approaches primarily based on immunotherapeutic approaches. Where once T1DM was viewed as a passive failure of pancreatic function, it is now recognized as the result of active immune-mediated destruction of insulin-producing β-cells. The transition was fueled by technological innovations such as autoantibody assays and HLA genotyping as well as by conceptual realignments that drew from immunology, genetics, and systems biology.

**Figure 1:**
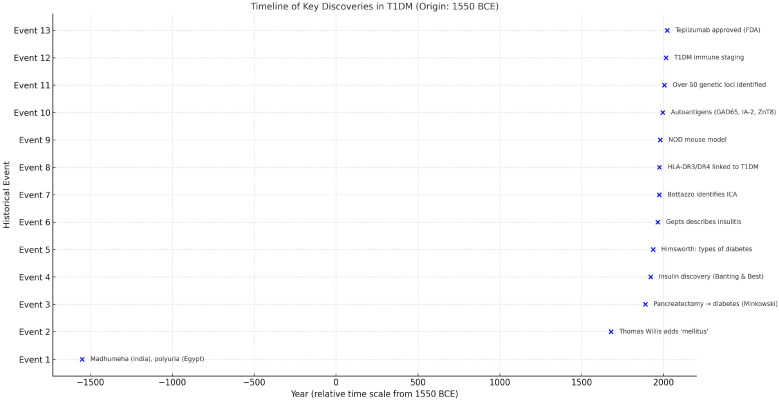
Timeline of key discoveries in the history of T1DM (origin: 1550 BCE).

In this review, we examine the chronological and epistemological transformation of T1DM. Beginning with ancient medical texts and early anatomical observations, we follow the progression through the insulin era, the recognition of disease heterogeneity in the mid-20th century, and finally its autoimmune origin that represented a radical conceptual shift in the 1970s. We then explore how the consolidation of immunological evidence led to new classification systems, prevention trials, and finally, disease-modifying therapies in the 21st century. Our analysis integrates classical and contemporary literature (see Figure 1 for a visual timeline of key discoveries in T1DM), including the recent comprehensive review by Herold et al., and considers how shifts in scientific instruments, institutional priorities, and interpretative frameworks have redefined not only how T1DM is treated, but what it is [1]. In doing so, we aim to contextualize T1DM as a model for understanding how diseases evolve in the collective imagination of science and medicine.

This timeline highlights 13 major milestones in the conceptual and scientific evolution of T1DM, spanning from early clinical descriptions in antiquity to modern immunogenetic discoveries and therapeutic breakthroughs. The horizontal axis represents the year of discovery, while the vertical axis lists the chronological order of events.

The conceptual foundation of immunotherapy in T1DM is deeply rooted in the historical observation of autoimmune-mediated β-cell destruction. During the late 20th century, experimental evidence accumulated from animal models such as the BB rat and the NOD mouse, which revealed that the development of diabetes was preceded by islet inflammation and infiltration by T lymphocytes [2,3]. These models provided an essential platform to test hypotheses regarding the role of autoreactive immune cells in T1DM and laid the groundwork for future therapeutic strategies aimed at restoring immune tolerance.

At that time, the idea of treating a metabolic disorder such as diabetes with immunosuppressive or immune-modulating agents was both pioneering and controversial. Yet it reflected a growing awareness that T1DM, far from being a purely endocrine condition, required immunological thinking and intervention [4]. Early trials using nonspecific immune-suppressants such as cyclosporine A generated cautious optimism by temporarily preserving β-cell function, despite notable toxicity and lack of long-term efficacy [5].

**Figure 2:**
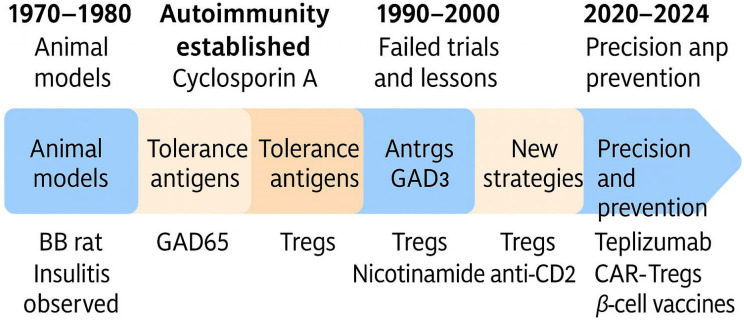
Timeline of key milestones in the development of immunotherapeutic strategies for T1DM.

Building upon these early insights, the field of T1DM immunotherapy has evolved through several distinct phases from antigen-specific tolerance induction and cytokine modulation to engineered cellular therapies and precision medicine [6]. These developments are not only scientific milestones but also markers of an ongoing shift in how autoimmunity is conceptualized and managed. A graphical timeline illustrating these key stages and milestones is shown in Figure 2, serving as a visual guide to the evolving landscape of T1DM immunotherapy.

This schematic highlights major phases and breakthroughs in T1DM immunotherapy, starting from early animal models in the 1970s (BB rat, insulitis observation), through the identification of autoimmunity and early specific or semi-specific tolerogenic approaches (GAD65, specific inhibitors of IFN-gamma and IL-1β, Tregs), and the use of cyclosporin A. The 1990s marked a period of unsuccessful clinical trials, followed by new strategies such as Tregs, nicotinamide, and anti-CD20 monoclonal antibodies. The most recent era (2020–2024) reflects precision approaches aimed at prevention and immune modulation, including Teplizumab, CAR-Tregs, and β-cell targeted vaccines.

## 2. Before Insulin: Diabetes in Antiquity and the Pre-Modern Era

The understanding of diabetes mellitus (and what would much later be divided into distinct subtypes) emerged slowly over millennia, shaped by observation, metaphor, and prevailing medical theories. Long before the biochemical or immunological underpinnings of the disease were even imaginable, diabetes was recognized and described based on its dramatic clinical features, most notably polyuria and wasting. Ancient medical traditions interpreted these symptoms through the lens of humoral imbalance or spiritual affliction, with no concept of endocrine or immune systems. Yet these early accounts laid the groundwork for a persistent misconception: that diabetes was a singular, purely metabolic condition. This historical framing, though clinically useful, would come to obscure the heterogeneous and often autoimmune nature of what is now called type 1 diabetes mellitus (T1DM). In this section, we trace the roots of diabetes through antiquity and early modern medicine, setting the stage for the later paradigm shifts that redefined its pathogenesis and treatment. The origins of what we now identify as T1DM trace back over three millennia. In the Ebers Papyrus (circa 1550 BCE), Egyptian physicians described polyuric syndromes marked by excessive urination, a hallmark of hyperglycemia [7]. Ancient Indian Ayurvedic texts referred to Madhumeha, or “honey urine,” reflecting an early recognition of glycosuria. The Greek physician Aretaeus of Cappadocia coined the term “diabetes” in the 2nd century CE, from the word siphon, to describe the seemingly uncontrollable flow of fluids through the body. In the 17th century, Thomas Willis famously added mellitus meaning “sweet” after tasting patients’ urine and noting its sugary flavor [8]. Despite such detailed phenomenology, the nature of the disease remained obscure. The humoral theory of disease prevailed, attributing symptoms to imbalances of bodily fluids. Even when anatomical studies emerged during the Renaissance, little was understood about the pancreas. Paracelsus, for example, observed crystalline substances in diabetic urine but could not link them to sugar or pancreatic pathology. It was not until the late 19th century that researchers like Étienne Lancereaux proposed a distinction between “diabète maigre” and “diabète gras,” hinting at heterogeneity, though without a molecular basis [9]. A major conceptual leap occurred in 1889, when von Mering and Minkowski removed the pancreas from dogs, inducing severe diabetes, a key demonstration that pancreatic secretions were essential to glucose homeostasis [10]. However, without a clear understanding of hormones, their work did not immediately resolve the disease’s mystery. In the early 20th century, treatment strategies focused on extreme caloric restriction, such as the Allen diet, which delayed death but led to starvation [11].

**Figure 3:**
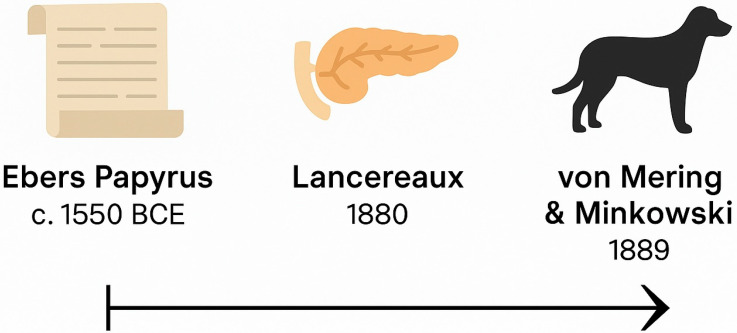
Timeline of key conceptual milestones in diabetes understanding before the discovery of insulin.

Crucially, this entire era framed diabetes as a uniform metabolic disorder. Neither distinction between types was made nor was the immune system considered relevant (see Figure 3 for a visual summary of key milestones in diabetes history prior to the discovery of insulin). The discovery of insulin has been a milestone and has dramatically revolutionized both the therapy and the prognosis of T1DM [12]. Initial histopathological clues such as occasional lymphocytic infiltration were either missed or dismissed [13]. Thus, the pre-insulin age laid a conceptual foundation that would both advance and constrain future thinking: diabetes was metabolic, not immunological, and singular, not heterogeneous.

Before 1921, a diagnosis of diabetes in children almost invariably led to death, often preceded by extreme emaciation and coma [11]. The discovery and therapeutic introduction of insulin by Banting and Best in 1921 dramatically reversed this prognosis, allowing patients to survive, gain weight, and live for decades [14,15]. However, this therapeutic breakthrough also entrenched a metabolic framework of disease understanding, which delayed the recognition of immune-mediated mechanisms for decades [1].

## 3. The Insulin Revolution: From Fatal Diagnosis to a Chronic Condition (1921–1950)

**Figure 4:**
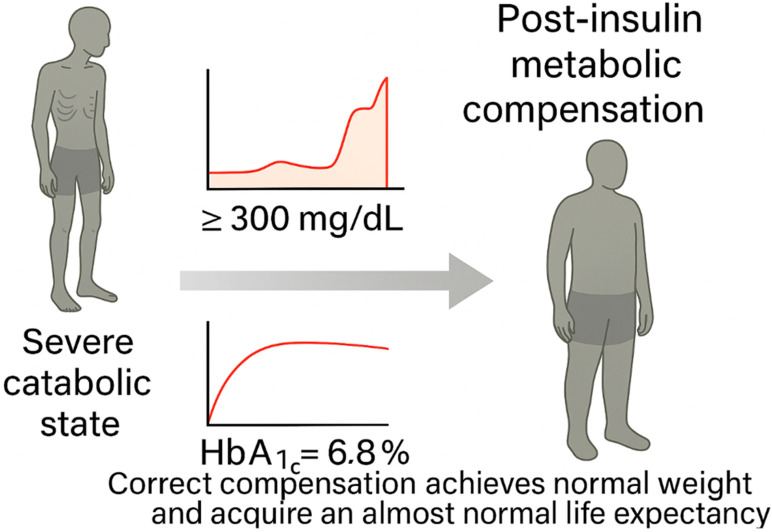
Conceptual representation of the clinical impact of insulin discovery in type 1 diabetes.

The discovery of insulin in the early 1920s represents one of the most dramatic shifts in the history of medicine [12]. In 1921, Canadian physician Frederick Banting and medical student Charles Best, working in J.J.R. Macleod’s laboratory at the University of Toronto, isolated a pancreatic extract that markedly reduced blood glucose in diabetic dogs [14]. In January 1922, they administered this extract, later refined and named insulin, to 14-year-old Leonard Thompson, marking the beginning of a therapeutic revolution [15]. Within a year, commercial production of insulin began through partnerships with Eli Lilly and Connaught Laboratories, and by 1923, Banting and Macleod had received the Nobel Prize [14]. While insulin saved lives and became a cornerstone of diabetes management, it also cemented the view of diabetes as a purely metabolic disorder, characterized by insulin deficiency and hyperglycemia (see Figure 4 for a conceptual depiction of the transformative clinical impact of insulin therapy on type 1 diabetes). This framework would dominate thinking for the next five decades [16,17].

During this era, no clear distinction was made between what are now classified as type 1 and type 2 diabetes. Although Harold Himsworth proposed the classification of insulin-sensitive and insulin-insensitive diabetes in 1936, this remained a metabolic differentiation, not an etiological one [16]. Autopsy studies occasionally showed lymphocytic infiltration of the islets, but these were largely dismissed as nonspecific [18].

Importantly, the overwhelming clinical success of insulin paradoxically suppressed further etiological inquiry, and [1] the therapeutic efficacy of insulin reinforced the metabolic model and delayed the integration of immune-based hypotheses (1). Even in the face of growing recognition of autoimmune diseases in the mid-20th century, such as Hashimoto’s thyroiditis and Addison’s disease, diabetes remained conceptually isolated from immunological thinking [19,20].

This period thus laid the groundwork for both progress and a fundamental change in medical framing. While it transformed diabetes care, it also entrenched assumptions that would only be challenged in the 1970s with the discovery of islet autoantibodies and the rediscovery of insulitis [18,21].

Before 1921, a diagnosis of diabetes in children almost invariably led to death, often preceded by extreme emaciation and comas. The discovery and therapeutic introduction of insulin by Banting and Best in 1921 dramatically reversed this prognosis, allowing patients to survive, gain weight, and live for decades. However, this therapeutic breakthrough also entrenched a metabolic framework of disease understanding, which delayed the recognition of immune-mediated mechanisms for decades.

## 4. Heterogeneity and the Pre-Autoimmune Puzzle (1950–1970)

In the mid-20th century, the growing availability of clinical data began to challenge the idea of diabetes mellitus as a homogeneous disorder. As Stefan Fajans & Conn in 1960 noted, “These diabetic subjects do not spontaneously present themselves to the physician or researcher; it is the investigator who must find them by testing asymptomatic relatives of known diabetic patients” [22].

Physicians observed striking differences in disease onset, progression, and therapeutic response across age groups. Pediatric patients typically presented with rapid onset of symptoms, profound insulin deficiency, and a high risk of ketoacidosis, while adult patients often showed a more indolent course, with varying degrees of insulin dependence [23]. Despite these differences, the notion of diabetes as a single metabolic entity persisted in textbooks and clinical practice [24].

The possibility of underlying heterogeneity remained underexplored in part because of the absence of mechanistic tools. Biomarkers for immune activity were nonexistent, and pancreatic histopathology was rarely performed post mortem. Nonetheless, isolated autopsy reports such as those later compiled by Gepts revealed the presence of lymphocytic infiltration in the islets of Langerhans, suggesting an inflammatory, perhaps immune-mediated process [18]. These findings, however, were often dismissed as nonspecific or artefactual [25].

Although other diseases such as Hashimoto’s thyroiditis, Addison’s disease, and pernicious anemia were increasingly understood as immune-mediated pathologies [19], the conceptual link between diabetes and autoimmunity remained tenuous. The pancreas was not yet viewed as a potential target of immune aggression, and diabetology remained largely under the influence of endocrine and metabolic frameworks [26].

Genetic studies were equally limited, though familial clustering of diabetes was observed, prompting speculation about inherited predisposition [27]. Without HLA typing or genome-wide tools, however, these hypotheses lacked confirmatory evidence [28]. Immunology and endocrinology, still distinct disciplines, had not yet converged in the study of diabetes.

The terminology used during this period further obscured disease subtypes. The labels “juvenile-onset” and “maturity-onset” diabetes, though descriptive, lacked pathophysiological clarity and reinforced the false assumption that diabetes was a temporally stratified but mechanistically uniform condition [29].

**Figure 5:**
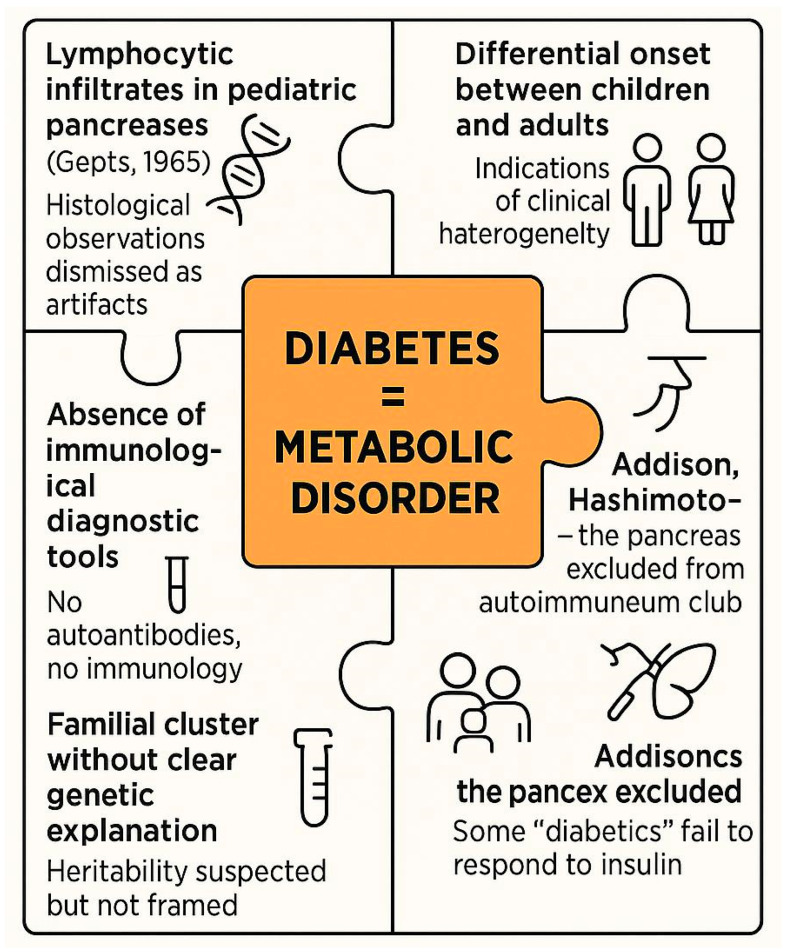
Overlooked signals and conceptual inertia before the autoimmune paradigm of type 1 diabetes (1950–1970).

This era, therefore, represents a critical liminal phase (see Figure 5). The signs of autoimmune involvement were present but uninterpreted, like puzzle pieces without a framework. Only in the 1970s, with the discovery of islet autoantibodies and the rise of immune-centric models, did these fragments begin to coalesce into a coherent immunological narrative [21].

Multiple lines of clinical and histological evidence, such as lymphocytic infiltrates, clinical heterogeneity and familial clustering, were present but unrecognized or misinterpreted due to the prevailing metabolic framework. These fragmented observations failed to coalesce into a coherent autoimmune hypothesis until the early 1970s.

## 5. The Autoimmune Paradigm Shift (1971–1976)

The period between 1971 and 1976 marks a pivotal turning point in the conceptual history of T1DM. It was during these years that the disease underwent a Kuhnian full-on change of lens from a metabolic deficiency to an autoimmune pathology [30]. As noted in a retrospective analysis, “the lack of understanding, in humans, of the role for the immune responses underlying β-cell destruction in general but, in particular, the absence of specific markers of autoimmunity, made it difficult to establish a direct link between immune mechanisms and the pathogenesis of type 1 diabetes” [31].

A central breakthrough came in 1974 when Bottazzo and colleagues published their discovery of islet cell antibodies (ICAs) in newly diagnosed diabetic children, especially those with polyglandular autoimmune syndromes [21]. This provided the first serological evidence of autoimmunity against pancreatic islets, fundamentally challenging the insulin-centric view of disease pathogenesis. Parallel to this, advances in histopathology had already begun to suggest a role for the immune system. Gepts (1965) had documented lymphocytic infiltration later termed “insulitis” in the pancreas of children who had died shortly after diagnosis, although these findings were initially regarded as incidental [18].

By mid-decade, accumulating evidence from both histological and immunological fronts began to align. Several groups replicated ICA detection and extended findings to broader populations, including first-degree relatives of patients [20]. The growing recognition of autoimmune polyendocrine syndromes, including Addison’s disease and Hashimoto’s thyroiditis, further bolstered the plausibility of a shared immune etiology [19,32,33].

The introduction of HLA typing provided additional weight. Studies by Cudworth and Woodrow (1975) demonstrated a significant association between juvenile-onset diabetes and specific HLA haplotypes, particularly DR3 and DR4, establishing a genetic–immune axis of susceptibility [34]. These insights were reinforced by later findings in monozygotic twins and population-level cohorts [35].

**Figure 6:**
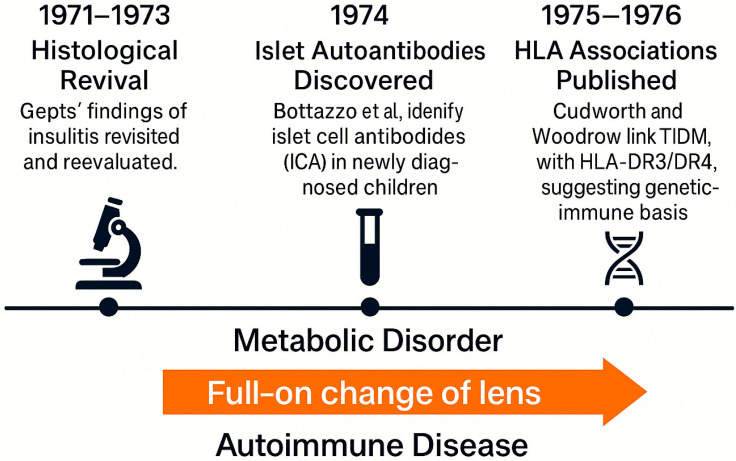
The autoimmune paradigm shift in type 1 diabetes (1971–1976).

Despite the strength of this emerging model, resistance from the clinical community was still observed. As Herold et al. highlight, many practitioners remained skeptical, anchored in the success of insulin therapy and unaware of immune-mediated mechanisms operating years before clinical onset [1]. The autoimmune model initially lacked therapeutic application; there was no intervention to “treat” autoimmunity, making it less attractive to practicing diabetologists (see Figure 6).

Nevertheless, this period crystallized the autoimmune hypothesis and redefined T1DM not merely as a hormonal deficiency but as an immune-mediated process. This paradigm shift was substantiated by the identification of islet autoantibodies and a strong association with specific HLA class II alleles, underscoring the immunogenetic basis of the disease [36]. Furthermore, the conceptualization of T1DM as a progressive, staged autoimmune disease emerged, delineating a sequence from asymptomatic autoimmunity to overt clinical diabetes, thereby providing a framework for early prediction and intervention strategies [37,38].

Building on these foundational discoveries, starting from the early 1980s, several experimental studies further elucidated the critical role that certain “hormones of the immune system”, named cytokines, played in the pathogenesis of the disease [39]. More specifically, several in vitro and in vivo studies generated solid proof of concept that a subset of proinflammatory cytokines such as interleukin (IL)-1 beta, tumor necrosis factor (TNF)-alpha and macrophage migration inhibitory factor (MIF) played an important pathogenic role in the pathogenesis of T1DM. Accordingly, it was shown “in vitro” that combined exposure to IFN-γ and IL-1β enhanced nitric oxide and TNF-α release from human fetal islets, contributing to β-cell cytotoxicity [40]. Subsequently, our group was the first to demonstrate the in vivo contribution of IL-1 beta, Interferon-gamma, IL-18 and macrophage migration inhibitory factors (MIFs) in BB rats, NOD mice, and/or mice made diabetic with multiple low doses of streptozotocin. We and others have also shown that treatment with anti-inflammatory cytokines such as IL-4 [41], IL-10 [42], IL-11 and IL-13 ameliorated the course of the disease in NOD mice [43,44]. This “in vitro” and “in vivo” evidence supported the pathogenetic concept that beta-cell destruction in T1DM may be mediated by the Thelper 1 (Th1) subset of T cells producing the proinflammatory cytokines IL-2 and IFN-gamma and counteracted by Th2 cells that produce anti-inflammatory cytokines such as IL-4, IL-10, and IL-13. More recent evidence has also suggested that the Th17 subset that produces IL-17 may also play an important role in the pathogenesis of T1DM [45]. This pathogenetic concept was further strengthened through the demonstration by ourselves and others that IFN-gamma-inducible IP-10 is increased in newly diagnosed T1DM patients and that IL-18 levels are increased in “prediabetic” individuals [46,47]. Several other abnormalities have been reported, often with conflicting results, in blood levels of several cytokines in newly diagnosed T1DM patients [48], which, as a whole, supports the concept that a dysregulated immune response takes place in the early stages of human T1DM. 

Together, these studies support the hypothesis that the proinflammatory cytokine milieu plays a crucial role in the onset and progression of T1DM and may represent a valuable target for immunomodulatory intervention. The translational relevance of “cytokinology” in T1DM pathogenesis stems from a recent study that demonstrates that downstream targeting with Baricitinib of JAK1 and JAK2 signaling, which is used by several cytokines, ameliorates the course of T1DM in newly diagnosed patients [49].

Key discoveries between 1971 and 1976, including the histological reevaluation of insulitis, the identification of islet autoantibodies, and the association of T1DM with HLA class II alleles, catalyzed a Kuhnian shift in understanding the disease as autoimmune rather than purely metabolic.

## 6. Consolidating Autoimmunity: From Experimental Models to Human Trials (1980–2000)

Following the paradigm shift in the 1970s, the two final decades of the 20th century saw the consolidation of T1DM as a prototypical autoimmune disease. Early findings such as the presence of islet cell autoantibodies and pancreatic insulitis had laid the groundwork, but it was in the 1980s and 1990s that these observations were mechanistically connected through a combination of animal models, human studies, and genetic insights [50].

The non-obese diabetic (NOD) mouse became an indispensable tool during this period. This animal model developed spontaneous autoimmune diabetes that mirrored human T1DM in both histology and immunopathogenesis [51,52]. As indicated above, NOD mice have proven essential to dissect the histological, cellular and biomolecular responses underlying T1DM and in vivo preclinical evaluation of novel immunotherapeutic approaches [53]. Autoantigen discovery was another cornerstone of this era. Beyond islet cell antibodies, specific autoantigens such as insulin itself, GAD65, IA-2, and ZnT8 were identified, allowing for improved stratification of disease risk [54,55].

Studies showed that the presence of multiple autoantibodies predicted progression to clinical diabetes, especially among genetically predisposed individuals [56,57].

This led to the formulation of a disease “staging” model based on immune markers, later refined in the 21st century.

Genetics also gained prominence. The association of T1DM with HLA-DR3 and DR4 alleles was confirmed in large-scale studies, and non-HLA genes such as INS, PTPN22, and CTLA4 were linked to immune regulation and disease susceptibility [58,59]. These findings demonstrated that T1DM was a complex polygenic disease, shaped by both immunogenetic and environmental interactions [60].

Clinical translation was more difficult. Trials using cyclosporin A showed some promise in preserving residual β-cell function but were marred by nephrotoxicity and relapse upon discontinuation [61,62]. Other immunotherapies, including oral insulin and nicotinamide, and the FusiDM trial by our own group failed to prevent disease onset in high-risk individuals [63,64,65,66].

**Figure 7:**
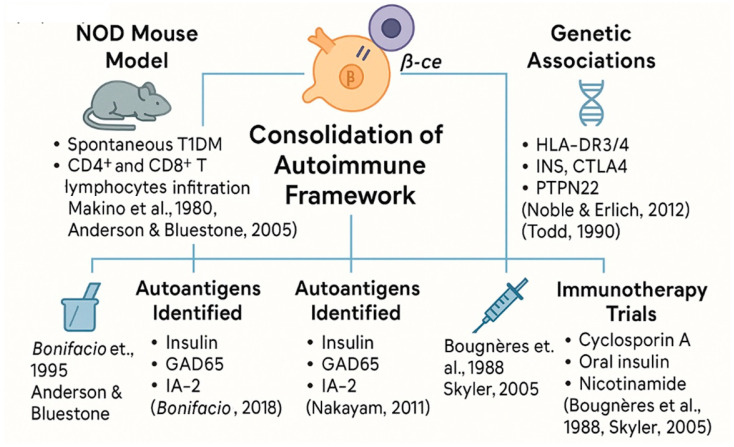
Experimental and clinical pillars that consolidated the autoimmune model of T1DM (1980–2000).

Despite these setbacks, this era solidified T1DM’s place in the autoimmune canon (see Figure 7). It marked a transition from observation to mechanistic exploration, setting the stage for precision immunology and interventional trials in the 21st century [1,67].

Spanning animal models, antigenic targets, genetic discoveries, and immunotherapy trials, this period established type 1 diabetes as a prototypical organ-specific autoimmune disease and laid the groundwork for translational immunology.

## 7. The Immunological Turn of the 21st Century: Biomarkers, Therapies, and Prevention (2000–2024)

The early 21st century marked a transition from descriptive immunology to translational immunotherapy in the management of T1DM. By this point, the autoimmune nature of the disease was well established, but researchers shifted their focus toward early prediction, immune staging, and disease interception. This “immunological turn” was underpinned by significant advances in genetics, bioinformatics, biomarker discovery, and immune modulation strategies [1,37,68].

Major efforts were directed toward defining precise immune phenotypes, discovering novel autoantibody and T-cell biomarkers predictive of disease progression [69,70] and developing preventive interventions targeting the presymptomatic stages of T1DM [71,72]. Advances in next-generation sequencing and systems immunology further accelerated the identification of molecular signatures associated with different stages of autoimmunity [1,73], facilitating stratified clinical trial designs and personalized immunotherapy approaches.

One of the most transformative developments was the identification of preclinical stages of T1DM based on serological and metabolic markers. The presence of multiple islet autoantibodies such as anti-GAD, IA-2A, IAA, and ZnT8A was found to predict near-certain progression to symptomatic disease, particularly in individuals with HLA-DR3/DR4 genotypes [38,74]. This led to the formalization of a three-stage model: Stage 1 (autoantibodies, normoglycemia), Stage 2 (dysglycemia), and Stage 3 (clinical onset) [37].

At the same time, genomic studies revealed more than 50 susceptibility loci beyond the HLA region, including INS, PTPN22, IL2RA, and IFIH1, helping refine risk stratification and deepening understanding of immune pathways involved in β-cell destruction [75,76]. This genomic insight supported the development of personalized risk scores and opened new avenues for prevention trials [77].

On the therapeutic front, immune interventions became increasingly targeted. The Anti-CD3 monoclonal antibody Teplizumab delayed progression from Stage 2 to Stage 3 in high-risk individuals [78]. In 2022, Teplizumab received FDA approval, the first drug ever authorized to delay the onset of T1DM, representing a watershed moment in the field (FDA, 2022). Other agents under investigation include Abatacept (CTLA4-Ig), low-dose IL-2, anti-CD20 (rituximab), and combination therapies [79].

In a more recent Phase II study, Teplizumab was also shown to exert beneficial effects when administered to patients with newly diagnosed T1DM [80].

Despite progress, major challenges remain. Not all at-risk individuals progress at the same rate, and not all therapies work equally well across age groups or immunophenotypes. Concepts like endotypes and immune resilience are being introduced to explain this variability and guide next-generation therapies [68].

**Figure 8:**
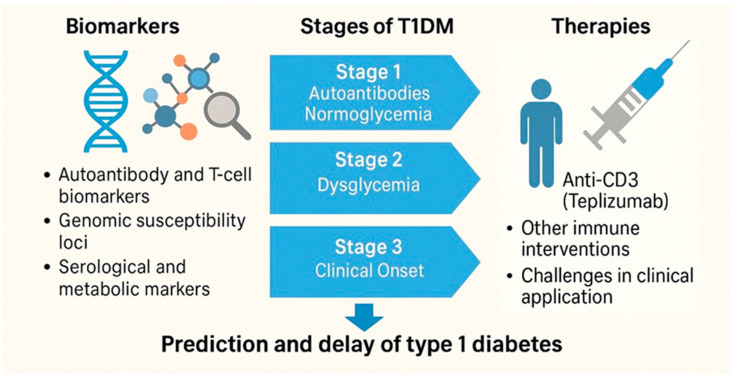
Key milestones in the immunological and translational turn of type 1 diabetes (2000–2024).

Today, T1DM is not only managed but preemptively studied. Longitudinal cohorts like TrialNet and TEDDY have redefined natural history research, while real-world implementation of screening and early therapy raises questions of ethics, access, and cost [81,82]. The 21st century has thus transformed T1DM from a fatal pediatric illness into a disease that can be monitored, anticipated, and at least in part modulated (see the key milestones in the immunological and translational turn of type 1 diabetes, Figure 8).

Biomarker discovery, disease staging, and immunointervention have redefined T1DM from a clinically reactive condition to one that can be predicted and modulated. The progression from risk identification to FDA-approved therapies marks a new phase in autoimmune precision medicine.

Despite promising advances, current immunotherapeutic approaches to T1DM face several critical limitations. As discussed, Teplizumab delays (but does not prevent) the progression from Stage 2 to Stage 3, with variable efficacy and no significant long-term impact on HbA1c in many patients [83]. Moreover, responders constitute only a subset of treated individuals, and repeat dosing often raises concerns about cost, chronic immunomodulation, and potential adverse effects.

Importantly, therapies targeting regulatory T cells (Tregs) including adoptive polyclonal transfer have proven safe but largely ineffective due to poor in vivo persistence and a lack of antigen specificity [84]. Recently, autologous engineered Tregs (e.g., GNTI-122) have shown promising preclinical activity, trafficking to the pancreas and suppressing islet-specific effector T cells [85]. Similarly, CAR-Treg strategies targeting islet antigens (e.g., GAD65, ENTPD3) represent an exciting frontier, though challenges remain in identifying optimal targets and ensuring long-term stability [86,87].

Future directions are therefore shifting towards combination and precision approaches: pairing antigen-specific tolerance with checkpoint blockade or metabolic support; developing engineered cellular therapies (CAR-Tregs, engineered FOXP3+ Tregs) with tailored homing and persistence [88]; optimizing delivery through nanoparticles or hydrogels to reduce systemic exposure; integrating vaccine-based β-cell antigen interventions for at-risk individuals; and exploring B-cell directed therapies (e.g., anti-CD20) as adjuncts in early-stage disease.

**Figure 9:**
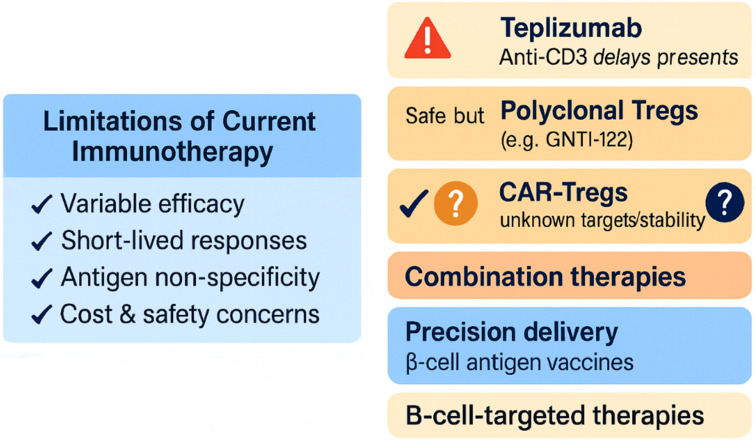
Current limitations and future directions in T1DM immunotherapy.

In summary, the immunotherapeutic paradigm is evolving from single-agent interventions to multifaceted, personalized regimens aimed at durable tolerance with minimized risk, ushering in an era of precision immunotherapy for T1DM (see the current limitations and future directions in T1DM immunotherapy in Figure 9).

Approved therapies like teplizumab delay but do not prevent disease onset and benefit only a subset of patients [83]. Regulatory T cell-based strategies remain limited by poor persistence and specificity [84]. Emerging approaches including CAR-Tregs, nanoparticle delivery, and antigen-specific vaccines aim to overcome these hurdles and shift toward personalized, durable immune tolerance [85,87,88].

## 8. The Contribution of Dismantled Immune Pathogenetic Concepts to the Diagnosis of T1DM: From Urine Testing to Seroimmunological and Biomolecular Analyses

The diagnosis of T1DM has undergone a profound transformation, evolving from rudimentary clinical observations to the current implementation of sophisticated immunological and genetic assays. Historically, the clinical recognition of diabetes was anchored in the detection of glycosuria, a method dating back to the 17th century when Thomas Willis described the sweet taste of diabetic urine. In the 19th century, chemical tests using Benedict’s or Fehling’s reagents enabled rudimentary quantification of glucose in urine, but lacked specificity and sensitivity. Blood glucose testing only emerged in clinical practice during the early 20th century, facilitated by the development of accurate enzymatic assays and the invention of portable glucose meters in the 1970s. Despite these advancements, T1DM remained indistinct from type 2 diabetes until the late 20th century, when immunological markers and HLA genotyping reshaped its clinical profile. The discovery of islet cell autoantibodies (ICAs) in the mid-1970s represented a pivotal moment, identifying T1DM as an autoimmune disease [21]. These antibodies were initially identified by indirect immunofluorescence techniques, revealing cytoplasmic staining patterns in pancreatic islets of deceased donors. Shortly thereafter, more specific autoantibodies were discovered, including anti-GAD65, anti-IA2, insulin autoantibodies (IAAs), and, later, ZnT8 autoantibodies, each providing increasing resolution for diagnosing preclinical and overt T1DM [38,89]. Modern diagnostic frameworks are grounded in the detection of two or more diabetes-associated autoantibodies, which predict progression to overt disease with high sensitivity and specificity [90]. These biomarkers are now routinely employed in longitudinal birth cohort studies such as DAISY, TEDDY, and DIPP to detect islet autoimmunity in genetically predisposed children. In such contexts, the presence of multiple antibodies implies an almost certain future diagnosis of T1DM [38]. Parallel to serological advances, HLA genotyping emerged as a cornerstone for stratifying genetic risk. The identification of the HLA-DR3/4-DQ2/8 heterozygous haplotype as a major genetic determinant helped identify neonates at greatest risk [91]. Today, HLA typing is employed in neonatal screening programs to preselect candidates for immunological surveillance. Though HLA risk haplotypes are necessary, they are not sufficient for disease development, highlighting the need for integrated diagnostic approaches. A further revolution occurred with the ability to measure beta-cell function through the quantification of C-peptide and stimulated insulin secretion. The disappearance of C-peptide over time reflects progressive beta-cell loss and is thus used to stage disease progression and evaluate therapeutic efficacy in interventional trials [92]. More recent developments include metabolomic profiling, such as the identification of distinct lipid signatures that precede seroconversion [93], although these remain research tools rather than clinical standards. The evolution of T1DM diagnostics reflects a deeper understanding of its pathophysiology, from a disease of sugar metabolism to one of targeted autoimmune beta-cell destruction. Today’s diagnostic algorithms integrate autoantibody panels, genetic susceptibility markers, and dynamic beta-cell functional assays. This paradigm shift enables not only earlier diagnosis, but also opens a window for pre-symptomatic intervention, marking the dawn of a predictive and preventative era in T1DM care (see Table 1).

A progressive evolution of diagnostic approaches for T1DM has occurred, from rudimentary symptom-based assessments and glycosuria detection in antiquity to the molecular profiling and immunological assays used in modern clinical practice. This timeline illustrates how advances in endocrinology, immunology, and genetics have refined diagnostic accuracy and allowed for earlier and more precise detection of disease onset.

## 9. Conclusions

The historical reconstruction of T1DM reveals not just the evolution of a clinical entity but the unfolding of a deeper transformation in biomedical understanding. From its earliest recognition as a polyuric wasting disease in antiquity, through its reclassification as a metabolic disorder in the insulin era, to its current status as a prototype of organ-specific autoimmunity, T1DM went through profound ontological shifts. These shifts reflect the interplay of observation, theory, technology, and institutional resistance, hallmarks of what Thomas Kuhn would describe as scientific revolutions.

The discovery of insulin in 1921 was both a triumph and a conceptual detour. While it saved lives and revolutionized treatment, it also entrenched a metabolic interpretation of the disease that delayed deeper inquiry into its cause. For decades, the focus on glycemic control overshadowed the heterogeneity in disease presentation, especially between children and adults, which hinted at divergent underlying mechanisms. These early clinical observations were dismissed or misinterpreted in the absence of tools capable of revealing the immune system’s role in β-cell destruction.

It was only in the 1970s, with the identification of islet cell autoantibodies and the histological detection of insulitis, that a new explanatory framework emerged. The autoimmune model not only redefined T1DM but placed it in a broader family of diseases involving aberrant immune responses to the self. This kind of classic shift was consolidated through the 1980s and 1990s via animal models (e.g., the NOD mouse), immunogenetic studies, and the identification of autoantigens. These advances allowed the disease to be dissected at the molecular level and situated within the emerging field of clinical immunology.

The 21st century has ushered in a new phase that is translational, preventive, and personalized. Through longitudinal studies and population screening, the natural history of T1DM has been mapped from its preclinical immune stages to full-blown metabolic failure. This temporal stratification has enabled the testing and approval of immunotherapies aimed at delaying onset, with teplizumab marking a historic milestone. Yet, for all its promise, immune modulation remains complex. Variability in patient response and the need for long-term efficacy call for nuanced models of disease progression, including emerging frameworks like endotyping.

Beyond the scientific advances, the story of T1DM is also a philosophical and institutional one. Its history illustrates how medical knowledge is not simply accumulated but restructured, how dominant paradigms resist change, and how new frameworks gain traction only when evidence, instrumentation, and theory converge. The shifts in T1DM understanding mirror broader tensions in biomedicine: between reductionism and systems thinking, between symptom control and etiological insight, and between immediate clinical application and long-term conceptual clarity.

In tracing this trajectory, T1DM serves as a microcosm of biomedical evolution and a case study in how diseases are not only discovered but constructed through the very tools and lenses with which we investigate them. The journey from glycosuria to genome-wide association studies, from insulin syringes to checkpoint modulators, reflects not only progress in treatment but a deeper reconfiguration of what it means to understand a disease.

As we look to the future, the challenge will be to sustain this integrative vision—to continue weaving together clinical, molecular, and philosophical perspectives. T1DM, once a mysterious killer of children, now stands as a sentinel disease at the frontier of immunology, prevention, and scientific imagination.

## 10. Epistemological Reflections on Paradigm Shifts in T1DM

The transformation of T1DM from a metabolic to an autoimmune disease offers a powerful lens through which to examine how biomedical paradigms evolve. This evolution, spanning centuries, exemplifies the dynamic interplay between empirical observation, technological innovation, and the philosophical frameworks that shape medical understanding. Thomas Kuhn’s theory of scientific revolutions is particularly instructive here. For decades, diabetes was entrenched within a metabolic paradigm, reinforced by therapeutic success with insulin and the lack of tools to explore immune mechanisms. It was only with the accumulation of anomalies, e.g., heterogeneity of clinical presentation, the discovery of islet cell autoantibodies, and evidence of insulitis, that a shift became both possible and necessary. This shift was not only empirical but epistemological: it redefined what constituted valid evidence, which disciplines held authority, and how causality was framed.

The case of T1DM also underscores the role of resistance in science. Clinicians and researchers working within the metabolic framework were initially reluctant to embrace an autoimmune model. This resistance illustrates how paradigms function not only as explanatory tools but also as institutional structures that gatekeep knowledge production. The eventual adoption of the autoimmune paradigm required more than new data; it required a reorganization of medical consensus.

Today, T1DM continues to evolve conceptually, as notions of disease staging, endotypes, and immune modulation introduce new layers of complexity. The history of T1DM serves as a reminder that scientific progress is rarely linear and that a full-on change of lens involves not only data but also deep shifts in perception, trust, and disciplinary boundaries.

## Figures and Tables

**Table 1:** Historical timeline of diagnostic tools in T1DM.

Period	Diagnostic Method	Key Features/Milestones
Antiquity–19th c.	Symptom-based observation	Diagnosis based on polyuria, weight loss, and “sweet-tasting” urine (e.g., ants attracted to urine).
Early 20th c.	Benedict’s and Fehling’s tests	Semi-quantitative detection of glycosuria; basic chemical assays for reducing sugars.
1921–1923	Discovery of insulin	Introduction of insulin therapy; stimulated need for more accurate diagnostic tools.
1940s–1960s	Blood glucose measurement	Use of colorimetric methods and enzymatic assays (e.g., glucose oxidase) in clinical practice.
1970s	C-peptide and insulin assays	Enabled discrimination between T1DM and T2DM; highlighted β-cell function status.
Late 1970s–1980s	Islet cell autoantibodies (ICAs)	First immunological biomarker of T1DM; foundation for autoimmune profiling.
1980s–1990s	Anti-insulin, anti-GAD, anti-IA-2 autoantibodies	Expanded autoantibody panel; allowed risk stratification in at-risk individuals.
1990s–2000s	HLA class II haplotype analysis	Genetic screening tools (e.g., DR3-DQ2/DR4-DQ8) identified high-risk genotypes.
2000s–2010s	ZnT8 autoantibodies; multiplex autoantibody assays	Improved sensitivity and specificity of early diagnosis and screening programs.
2010s–Present	Metabolomics and immune-cell profiling	Identification of pre-autoimmune metabolic signatures and autoreactive T-cell assays.
Present–Future	Multi-omics, AI-driven predictive algorithms	Integration of genomics, proteomics, and machine learning for personalized risk prediction.
